# Male Gender and Normal Trochlear Anatomy Are Associated with a Higher Risk of Osteochondral Fracture Following Patellar Dislocation: A Retrospective Review of 261 Skeletally Mature Patients

**DOI:** 10.3390/jcm14228235

**Published:** 2025-11-20

**Authors:** Johannes Rüther, Markus Geßlein, Michael Millrose, Maximilian Willauschus, Jonas Beck, Niklas Engel, Andreas Kopf, Hermann Josef Bail, Lotta Hielscher

**Affiliations:** 1 Department of Orthopedics and Traumatology, Paracelsus Medical University, 90471 Nuremberg, Germany; 2 Department of Trauma Surgery and Sports Medicine, Garmisch-Partenkirchen Medical Center, 82467 Garmisch-Partenkirchen, Germany; 3 Core Facility Biostatistics, Paracelsus Medical University Salzburg, 5020 Salzburg, Austria

**Keywords:** patellar dislocation, osteochondral fracture, trochlear dysplasia, patellofemoral alignment, MRI

## Abstract

**Background**: Osteochondral fractures (OCFs) following acute patellar dislocation significantly influence treatment decisions and long-term prognosis, yet reliable risk stratification remains elusive. This study aimed to identify demographic and trochlear morphology predictors of fracture occurrence in a large patient cohort. **Methods**: This retrospective analysis included 261 skeletally mature patients with acute patellar dislocation from 2000 to 2024 (mean age 24 ± 10 years, 59% male), excluding those with previous knee surgery, additional injuries, or skeletal immaturity. A comprehensive MRI assessment evaluated trochlear morphology (Dejour classification, sulcus angle, trochlear groove depth, and facet measurements) and patellofemoral alignment parameters (TT-TG distance, Q-angle, and congruence angle). Multivariate logistic regression identified independent risk factors for the development of osteochondral fractures. **Results**: Osteochondral fractures occurred in 133 patients (51% [of those undergoing MRI evaluation]). Male gender emerged as the strongest predictor (OR 2.38, 95% CI: 1.30–4.42, *p* = 0.005), followed by right-sided dislocation (OR 2.33, 95% CI: 1.21–4.58, *p* = 0.013). Notably, higher-grade trochlear dysplasia (Dejour Grades 3–4) was associated with lower fracture rates, being more common in non-fracture patients (27% vs. 10%, *p* = 0.003). Trochlear condyle asymmetry was also significant (OR 1.14, *p* = 0.004). Most patellofemoral alignment parameters, including TT-TG distance and Q-angle, showed no significant predictive value. **Conclusions**: Male gender and right-sided dislocation are associated with higher rates of osteochondral fracture after patellar dislocation. Patients with normal trochlear anatomy have higher fracture rates than those with severe dysplasia. These findings suggest that demographic factors and trochlear morphology should be considered in the diagnostic workup of acute patellar dislocations, though prospective validation is needed before implementing screening protocols.

## 1. Introduction

Acute patellar dislocation represents one of the most common knee injuries in adolescents and young adults, occurring in 23.2 per 100,000 individuals annually [1,2,3]. The injury predominantly affects individuals during the second and third decades of life, with peak incidence observed in the 10–17 age group, particularly among those engaged in sports activities [1,2].

Osteochondral fractures (OCFs) represent a critical complication of patellar dislocation, occurring in more than half of cases, depending on the imaging modality employed [4,5]. These fractures vary significantly in their presentation, ranging from small cartilaginous fragments to large osteochondral fragments involving substantial portions of the articular surface. The detection rate of OCF has increased substantially with the widespread adoption of MRI as the standard imaging modality, as many fractures remain occult on conventional radiography [4,5]. These injuries significantly impact both immediate treatment decisions and long-term prognosis, influencing surgical planning and rehabilitation protocols [6].

OCF frequently requires surgical intervention, with untreated defects predisposing to early-onset patellofemoral arthritis [7,8,9,10]. Current risk stratification approaches remain inconsistent and largely empirical [1]. Traditional assessment focuses on trochlear morphology through Dejour classification and patellofemoral alignment parameters, including TT-TG distance [1,11,12]. However, the relationship between these anatomical parameters and acute osteochondral fracture risk remains poorly defined. Some authors propose that severe dysplasia may protect against OCF by permitting dislocation with less traumatic force, though evidence for this hypothesis is limited [6,13,14].

Previous studies have been limited by sample sizes typically under 100 patients, heterogeneous populations, and inconsistent imaging protocols [6,13,14]. Recent consensus statements have emphasized the need for better risk stratification tools and standardized treatment approaches for first-time patellar dislocations [15,16]. The 2025 ESSKA consensus considers MRI evaluation mandatory for assessing predisposing factors and detecting osteochondral lesions after first-time dislocations [15]. The present study addresses these limitations with a large, homogeneous cohort of 261 patients using standardized MRI protocols and comprehensive morphological assessment.

The primary aim of this study was to identify demographic and trochlear morphology risk factors for osteochondral fracture development following acute patellar dislocation in a large patient cohort using standardized MRI-based assessment and multivariate statistical modeling. Secondary aims included evaluating the relationship between trochlear dysplasia severity and acute fracture risk and determining the predictive value of patellofemoral alignment parameters. This study hypothesized that patients with severe trochlear dysplasia would have lower OCF rates than those with normal anatomy due to lower-energy dislocation mechanisms.

## 2. Materials and Methods

### 2.1. Study Design and Patient Selection

The primary outcome was the presence or absence of osteochondral fracture identified on MRI, defined as any cartilaginous or osteochondral fragment, regardless of size, visible on MRI sequences.

This retrospective study included 296 patients with acute patellar dislocation between 2000 and 2024 treated at a Level I trauma center serving a population of approximately 1.5 million inhabitants in the metropolitan region. All patients presenting with suspected or confirmed patellar dislocation during the study period were screened for eligibility. Of the initial 296 patients, 35 were excluded due to missing MRI data (*n* = 18), incomplete demographic data (*n* = 9), bilateral involvement (*n* = 5), and loss to follow-up (*n* = 3), leaving 261 patients for analysis.

Exclusion criteria were as follows: additional injuries (defined as concurrent ligamentous, meniscal, or other bony injuries to the ipsilateral knee); history of patellar dislocation or previous knee surgery; chronic instability; patients without MRI diagnostics; skeletal immaturity (open physis) to ensure homogeneous biomechanical conditions, as pediatric patients have different injury patterns; inflammatory arthropathies; and neuromuscular disorders affecting the lower extremity.

All included cases were unilateral dislocations.

### 2.2. Imaging Protocol and Analysis

All MRI examinations were performed using 1.5 T or 3.0 T scanners according to standardized knee protocols as part of routine diagnostic protocol for acute patellar dislocations, following current consensus recommendations [15]. The imaging protocol included the following sequences: sagittal T1-weighted, coronal and axial proton density-weighted with fat saturation, and axial T2-weighted sequences. All imaging was performed within 7 days of the acute dislocation event.

MRI analysis assessed trochlear morphology, including Déjour classification, sulcus angle, congruence angle, trochlear facet asymmetry, medial and lateral trochlear facet lengths, trochlear groove depth, trochlear condyle asymmetry, lateral trochlear angle inclination, and lateral trochlear indices. Specific measurement techniques were as follows: The sulcus angle was measured on axial images 3 cm above the femorotibial joint line, using the deepest point of the trochlear groove as the vertex. Trochlear groove depth was measured as the perpendicular distance from the deepest point of the groove to a line connecting the highest points of the medial and lateral condyles. Medial and lateral trochlear facet lengths were measured from the anterior edge to the posterior extent of each condyle on sagittal images. The Déjour classification was assessed on true lateral radiographs when available and confirmed on sagittal MRI sequences. Trochlear condyle asymmetry was calculated as the ratio of medial to lateral condyle height.

Patellofemoral alignment parameters included TT-TG distance, TT-PCL distance, and Q-angle measurements. The TT-TG distance was measured as the distance between the tibial tuberosity and the deepest point of the trochlear groove on axial images, using a standardized technique with perpendicular lines from each landmark to a reference line through the posterior femoral condyles. The TT-PCL distance was similarly measured using the posterior cruciate ligament insertion as the medial reference point. The Q-angle was calculated from the intersection of lines drawn from the anterior superior iliac spine to the patella center and from the patella center to the tibial tuberosity.

Osteochondral fragment characteristics were documented, including fragment number, size (largest dimension in any plane), location (patellar, trochlear, or both), and presence of associated bone marrow edema patterns.

### 2.3. Inter-Observer Reliability

Two orthopedic surgeons performed MRI analysis separately to increase inter-observer reliability. Both observers had >5 years of experience in musculoskeletal imaging interpretation and were blinded to patient outcomes and clinical information beyond the imaging studies. Measurements were performed independently using standardized templates and measurement tools within the PACS system (Picture Archiving and Communication System).

### 2.4. Statistical Analysis

Statistical analysis included descriptive statistics, univariate comparisons, and logistic regression modeling. Continuous variables were tested for normality using the Shapiro–Wilk test. Normally distributed continuous variables (age, sulcus angle, congruence angle, trochlear facet asymmetry, medial and lateral trochlear facet lengths, trochlear condyle asymmetry, lateral trochlear angle inclination, Q-angle, TT-TG distance, and TT-PCL distance) were compared between fracture and non-fracture groups using unpaired *t*-tests. Non-normally distributed variables (trochlear groove depth) were analyzed using Mann–Whitney U tests. Categorical variables (gender, side, and Déjour classification grades) were compared using chi-square tests.

Multivariate logistic regression analysis was performed to identify independent predictors of osteochondral fracture occurrence. Variables for the logistic regression model were selected based on (1) clinical relevance from prior literature, (2) statistical significance in univariate analysis (*p* < 0.1), and (3) absence of multicollinearity. The inclusion of laterality (right vs. left) was based on biomechanical literature suggesting dominant-limb differences in injury patterns.

We modeled the binary outcome osteochondral fracture (OCF: Yes/No) using multivariable logistic regression. Candidate predictors included demographic factors (age, sex, and side) and MRI metrics. To improve interpretability and numerical stability, ratio-type MRI indices (e.g., Insall–Salvati, modified IS, Blackbourne–Peel, Canton–Deschamps, patella–trochlear index, trochlear facet/condyle asymmetry, and lateral trochlear index) were rescaled by 100; hence, odds ratios (ORs) reflect the effect per 1 percentage point increase in the index. Since the Déjour classification is ordered and contains sparse upper categories, it was entered as a single ordinal score (per grade increase).

Model coefficients are reported as ORs with 95% confidence intervals and *p*-values. Complete case analysis, inspected multicollinearity (VIF) among correlated MRI indices, and considered stepwise selection as a sensitivity check. Discrimination was summarized with the area under the ROC curve (AUC), and calibration was assessed using bootstrap calibration. Statistical significance was set at *p* < 0.05 for all analyses.

### 2.5. Ethical Considerations

The study protocol was approved by the institutional ethics board (IRB-2022-001 issued on 17 February 2022). The study was conducted in accordance with the Declaration of Helsinki and all applicable local regulations. Given the retrospective nature of the study, the ethics board granted a waiver of informed consent for the use of de-identified data. Patient confidentiality was maintained throughout the study, with all data stored on secure, password-protected servers accessible only to the research team.

## 3. Results

### 3.1. Patient Demographics and Baseline Characteristics

From the initial 296 patients, a total of 261 patients were finally included in the analysis. The mean age was 24 ± 10 years (median 21 years, IQR 16–30).

### 3.2. Osteochondral Fracture Prevalence and Distribution

Osteochondral fractures were identified in 133 patients (51%) following acute patellar dislocation, while 128 patients (49%) showed no evidence of fractures. Among patients presenting with osteochondral fractures, the distribution of osteochondral fragment numbers was as follows: single fragment in 69 patients (52%), two fragments in 29 patients (22%), three fragments in 9 patients (6.8%), four fragments in 2 patients (1.5%), and six fragments in 24 patients (18%).

The mean defect size of the fragments was 3.17 ± 3.19 cm (median 2.00 [1.00, 4.00]). The wide standard deviation reflects substantial heterogeneity in fracture severity, ranging from small chondral fragments to large osteochondral fragments.

Regarding fragment origin, among patients with fractures, 57 (44%) originated from retropatellar cartilage without distinction between medial and lateral facets due to imaging limitations, 51 (39%) from femoral, 22 (17%) from femoral and retropatellar cartilage, and 1 (0.8%) from tibial cartilage.

### 3.3. Univariate Comparisons

Univariate analysis revealed several significant differences between fracture and non-fracture groups (Table 1). Gender demonstrated the strongest association, with males representing 71% of fracture patients compared to only 45% of non-fracture patients (*p* < 0.001). Similarly, right-sided dislocations occurred in 43% of fracture cases versus 25% of non-fracture cases (*p* < 0.01). Regarding trochlear morphology, the Dejour classification showed a significant inverse relationship with fracture risk (*p* = 0.003). Notably, severe dysplasia (Grades C and D) was present in 27% of non-fracture patients but only 10% of fracture patients. Lateral trochlear facet length was significantly greater in fracture patients (22.4 ± 3.4 mm vs. 21.3 ± 3.7 mm, *p* = 0.021), while trochlear condyle asymmetry showed minimal but statistically significant differences (*p* < 0.05). Most patellofemoral alignment parameters, including TT-TG distance (13.3 ± 4.9 mm vs. 13.1 ± 5.6 mm, *p* = 0.743) and Q-angle (7° ± 16° vs. 4° ± 10°, *p* = 0.543), showed no significant differences between groups.

### 3.4. Logistic Regression Analysis

**Figure 1:**
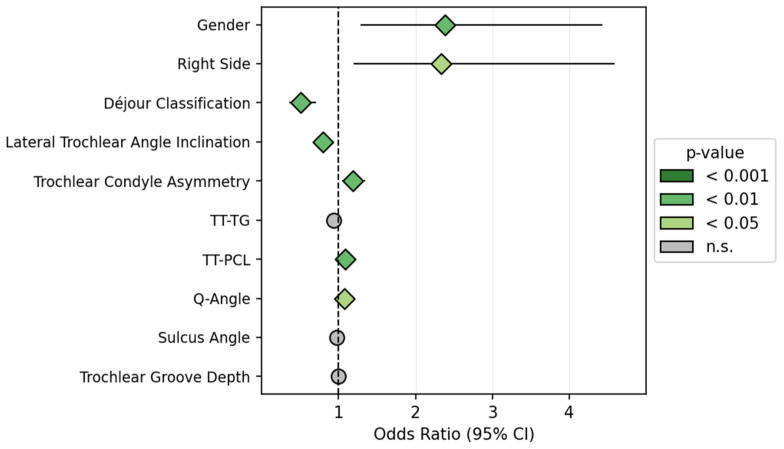
Logistic Regression–Forest Plot of Odds Ratios.

The logistic regression analysis demonstrates that demographic factors serve as the primary predictors of osteochondral fracture development, while most radiological parameters show limited predictive value in the multivariate model. Dejour classification, gender, and right side seem to have a relevant impact on the odds ratio (OR). The final multivariate model achieved good discrimination with an area under the ROC curve (AUC) of 0.74 (95% CI: 0.68–0.80), indicating moderate to good predictive performance. Calibration analysis demonstrated acceptable agreement between predicted and observed fracture probabilities across risk deciles. All other measurements did not affect the OR (Figure 1, Table 2).

## 4. Discussion

The present study demonstrates that male gender (OR 2.38, *p* = 0.005) and right-sided dislocation (OR 2.33, *p* = 0.013) represent the primary risk factors for osteochondral fractures, while patients with normal or low-grade trochlear dysplasia demonstrated higher fracture rates than those with severe dysplasia (10% vs. 27% in non-fracture patients, *p* < 0.001). Trochlear condyle asymmetry showed significance as a risk factor. Most patellofemoral alignment parameters showed limited predictive value. These findings expand conventional assumptions about the relationship between anatomical risk factors and acute traumatic complications, suggesting that risk stratification for OCF should focus on trauma, demographic, and biomechanical factors as well as static anatomical measurements [4].

The predominance of isolated patellar and femoral lesions rather than combined injuries in our cohort suggests distinct injury mechanisms, consistent with previous pathoanatomic studies. Most dislocations appear to generate focal impact injuries rather than diffuse cartilage damage, which would implicate surgical planning.

The male predominance may reflect biomechanical differences: greater quadriceps strength potentially generates higher forces during dislocation, and skeletal geometry variations between genders may influence injury mechanisms [1]. Higher activity levels in young males may contribute to more severe initial trauma as well [1]. Previous studies have documented significant gender differences in knee biomechanics, including variations in Q-angle, muscle activation patterns, and landing mechanics. Additionally, differences in bone density, cartilage thickness, and hormonal influences on collagen structure may contribute to varying fracture susceptibility between genders. While lower bone density in females might theoretically increase fracture risk, the findings in this study suggest that biomechanical factors such as impact force may override bone density effects. These findings align with recent studies examining patellar morphology and OCF risk. Zhou et al. (2024) demonstrated that specific patellar morphological features, particularly Wiberg angle and index, are independent risk factors for OCF occurrence [17]. This supports our observation that anatomical variations significantly influence fracture risk beyond traditional alignment parameters.

The gender effect persisted even after controlling for age and anatomical factors in our multivariate model, suggesting an independent biological contribution to fracture risk [2]. Understanding these gender-specific risk patterns has important clinical implications. Emergency physicians and orthopedic surgeons should maintain heightened suspicion for OCF in male patients, especially with high-energy trauma and in the absence of obvious anatomical risk factors. This awareness may influence decisions regarding imaging protocols and early intervention strategies.

Right-leg dominance in most individuals may lead to different injury mechanisms during sports or daily activities [2]. Approximately 85–90% of the population exhibits right-leg dominance for tasks such as kicking and jumping. This biomechanical preference may result in different loading patterns and muscle activation strategies between limbs during activities that predispose to patellar dislocation. The finding that right-sided dislocations carry higher fracture risk suggests that dominant-leg dislocations may occur under different circumstances than non-dominant-leg injuries. However, in this study, injury mechanism data was not collected, hence limiting our ability to confirm these biomechanical hypotheses.

Patients with normal trochlear anatomy showed higher fracture rates than those with severe dysplasia. An anatomically correct trochlear requires significantly more acting forces to lead to patella dislocation; when this occurs, the required force increases, concurrent with osteochondral injury likelihood [5]. Severe dysplasia allows lower-energy dislocations due to structural malformation, potentially reducing acute fracture risk despite chronic instability [6,11].

This phenomenon can be understood through a biomechanical framework. Normal trochlear morphology provides substantial bony constraint to patellar motion, hence requiring considerable force to overcome these stabilizing structures [11]. When dislocation does occur in the presence of physiologic anatomy, it implies that the applied forces were sufficient to overcome the bony and ligamentary barriers, suggesting significant mechanical force [5]. In contrast, patients with severe dysplasia (Dejour C and D) lack adequate bony containment and may experience recurrent dislocations with relatively minor trauma that does not generate sufficient shear forces to produce osteochondral fractures [6].

The clinical implications of this finding are profound. The 2025 ESSKA consensus considers MRI mandatory for evaluating predisposing factors and detecting osteochondral lesions after first-time dislocations [15]. The consensus also notes that osteochondral lesions ≥ 1 cm^2^ in the patellofemoral contact area should be repaired [16]. Patients presenting with first-time dislocation and normal radiographic findings should not be considered low risk for complications [6,18].

Clinical Implications

While the findings in this study suggest that male patients with normal trochlear anatomy may benefit from heightened clinical suspicion for OCF, routine screening protocols cannot be recommended based on this retrospective single-center study alone. Parikh et al. (2024) developed consensus guidelines recommending initial radiographs and selective MRI for first-time dislocations and suggested surgical treatment for concomitant osteochondral fractures [19]. The decision for MRI should remain individualized, considering clinical presentation, physical examination findings, and resource availability. Recent advances in surgical techniques for patellofemoral instability, as reviewed by Hinckel et al. (2024) [20], emphasize the importance of patient-specific approaches combining soft tissue and bony procedures based on individual risk factors.

Limitations

This study has several limitations. The retrospective design introduces potential selection bias. Patients who did not undergo MRI evaluation were excluded from analysis, potentially creating a selection bias toward more severely symptomatic individuals or those with higher clinical suspicion for fracture. Additionally, practice patterns regarding MRI utilization likely evolved over the 24-year study period, with earlier cases possibly undergoing less comprehensive imaging evaluation.

The 24-year timeframe spans technological changes in imaging and surgical techniques [4,13]. MRI technology, sequence protocols, and field strengths evolved considerably during this period, potentially affecting fracture detection rates and measurement precision [4]. Similarly, clinical awareness of OCF as a complication of dislocation has increased over time, possibly influencing imaging interpretation and documentation practices.

Binary fracture classification may oversimplify the injury severity spectrum [7]. This analysis categorized patients as having OCF present or absent, without detailed characterization of fracture size, depth, or location in the multivariate model. This simplification was necessary for statistical modeling but may obscure important relationships between specific anatomical factors and particular fracture patterns [5].

The term “high-energy trauma” used in the analysis was not directly measured but rather inferred from the presence of OCF in patients with normal anatomy, representing a limitation in mechanistic interpretation. Data on injury mechanisms was not collected, limiting the ability to confirm the proposed biomechanical explanations for the laterality effect.

Exclusion of patients with incomplete data may have introduced systematic bias. While no a priori sample size calculation was performed, post hoc analysis showed adequate power (0.82) for detecting the main effects observed.

Future Directions

Prospective multicenter studies with standardized protocols would strengthen findings. Prospective enrollment would eliminate selection biases inherent in retrospective review and allow standardized imaging protocols, measurement techniques, and outcome assessment [6,14]. Multicenter collaboration would increase sample size and geographic diversity, improving generalizability [13]. Future studies should consider contralateral imaging for comparison.

Investigation of anatomic values and fracture location, size, and long-term outcomes could refine diagnostics and therapy [7,9]. Future studies should analyze relationships between specific anatomical parameters and fracture characteristics such as location (patellar versus trochlear), size categories, and chondral versus osteochondral involvement [5]. Long-term follow-up studies correlating acute fracture characteristics with subsequent arthritis development, recurrent instability, and functional outcomes would inform treatment algorithms [9,10].

Development of clinical decision tools incorporating demographic and trochlear morphology factors could improve risk stratification [6,13]. Validated scoring systems combining multiple risk factors could guide imaging decisions, triage, and treatment planning. Machine learning approaches incorporating demographic, anatomical, and mechanistic variables might further optimize prediction models [14].

## 5. Conclusions

Male gender and right-sided dislocation are associated with higher osteochondral fracture rates after acute patellar dislocation. Patients with physiologic trochlear anatomy show higher fracture rates than those with severe dysplasia. These findings may help clinicians identify patients at risk for OCF, though prospective validation is needed before implementing systematic screening protocols. The decision for advanced imaging should remain individualized, considering clinical presentation and available resources.

## Figures and Tables

**Table 1:** Patient Characteristics and Statistical Comparison.

Characteristics	Category	No Fracture (n = 128)	Fracture (n = 133)	Overall (n = 261)	*p*-Value	Statistical Test
**DEMOGRAPHIC** **CHARACTERISTICS**						
Age (years, ±SD [range])		24 ± 10 [17–30]	23 ± 10 [16–27]	24 ± 10 [16–30]	0.542	*t*-test
Gender (n, %)	Female: Male:	70 (55%) 58 (45%)	38 (29%) 95 (71%)	108 (41%) 153 (59%)	**<0.001**	X^2^ = 18.34
Side (n, %)	Left: Right:	96 (75%) 32 (25%)	76 (57%) 57 (43%)	172(66%) 89 (34%)	**<0.01**	X^2^ = 9.26
**FRACTURE** **CHARACTERISTICS**						
Defect Size (cm, ±SD [range])		-	3.2 ± 3.2 [1.0–4.0]	-	-	-
**TROCHLEAR** **MORPHOLOGY (MRI)**						
Déjour Classification (n, %)	No dysplasia:	39 (30%)	63 (47%)	102 (39%)	**0.003**	X^2^ = 16.52
	Grade A:	18 (14%)	27 (20%)	45 (17%)		
	Grade B:	37 (29%)	30 (23%)	67 (26%)		
	Grade C:	27 (21%)	12 (9.0%)	39 (15%)		
	Grade D	7 (5.5%)	1 (0.8%)	8 (3.1%)		
Sulcus angle (°, ±SD)		150 ± 12	147 ± 10	148 ± 11	0.081	*t*-test
Congruence Angle (°, ± SD)		−1 ± 37	5 ± 22	2 ± 30	0.125	*t*-test
Trochlear Facet Asymmetry (±SD)		0.6 ± 0.2	0.6 ± 0.2	0.6 ± 0.2	0.689	*t*-test
Medial Trochlear Facet Length (mm, ±SD)		12.7 ± 2.9	13.3 ± 2.9	13.0 ± 2.9	0.162	*t*-test
Lateral Trochlear Facet Length (mm, ±SD)		21.3 ± 3.7	22.4 ± 3.4	21.9 ± 3.6	**0.021**	*t*-test
Trochlear Groove Depth (mm, ±SD)		5.1 ± 1.6	10.5 ± 60.8	7.9 ± 43.9	0.823	Mann–Whitney U
Trochlear Condyle Asymmetry (±SD)		1.0 ± 0.03	1.0 ± 0.03	1.0 ± 0.03	**<0.05**	*t*-test
Lateral Trochlear Angle Inclination (°, ±SD)		16.4 ± 4.5	15.5 ± 4.4	15.9 ± 4.5	0.378	*t*-test
**PATELLOFEMORAL ALIGNMENT**						
Q-angle (°, ±SD)		4 ± 10.4	7 ± 16.1	6 ± 14.2	0.543	*t*-test
TT-TG Distance (mm, ±SD)		13.1 ± 5.6	13.3 ± 4.9	13.2 ± 5.3	0.743	*t*-test
TT-PCL Distance (mm, ±SD)		24.2 ± 9.3	25.4 ± 10.1	24.9 ± 9.6	0.345	*t*-test

Data presented as mean ± SD, median [IQR], or n (%). Bold values indicate statistical significance, X^2^ test including Cohen’s effect size.

**Table 2:** Logistic Regression results.

Predictor	OR (95% CI)	*p*-Value
Gender	2.38 (1.3–4.42)	**0.005**
Right Side	2.33 (1.21–4.58)	**0.013**
Déjour Classification	0.51 (0.37–0.70)	**<0.001**
Lateral Trochlear Angle Inclination	0.80 (0.71–0.90)	**<0.001**
Trochlear Condyle Asymmetry	1.19 (1.06–1.34)	**0.004**
TT-TG	0.94 (0.88–1.01)	0.077
TT-PCL	1.09 (1.03–1.15)	**0.001**
Q-Angle	1.08 (1.03–1.13)	**0.028**
Sulcus Angle	0.98 (0.94–1.01)	0.157
Trochlear Groove Depth	1.00 (1.00–1.00)	0.094

Data are presented as Odds Ratio (OR) between groups of patients with and without OCF and the 95% confidence interval, as well as the *p*-value for statistical significance, bold values indicate statistical significance (*p* < 0.05).

## Data Availability

The original contributions presented in this study are included in the article. Further inquiries can be directed to the corresponding author.
